# Influence of Treadmill Design on Gait: Does Treadmill Size Affect Muscle Activation Amplitude? A Musculoskeletal Calculation With Individualized Input Parameters of Gait Analysis

**DOI:** 10.3389/fneur.2022.830762

**Published:** 2022-03-02

**Authors:** Matthias Woiczinski, Carolin Lehner, Thekla Esser, Manuel Kistler, Monica Azqueta, Johannes Leukert, Leandra Bauer, Eduard Kraft

**Affiliations:** ^1^Department of Orthopaedics and Trauma Surgery, Musculoskeletal University Center Munich (MUM), University Hospital, LMU Munich, Munich, Germany; ^2^Chair of Epidemiology, Department of Sport and Health Sciences, Technical University of Munich, Munich, Germany

**Keywords:** gait, treadmill, muscle modeling, healthy subject, geriatric (aging)

## Abstract

With increasing age, gait changes often occur, leading to mobility problems and thus a higher risk of falling. Interest in training at home or at retirement homes has led to the development of “mobile treadmills.” A difference in treadmill surface length may influence walking parameters (i.e., step length) and therefore may affect muscle activation. This led to the question: Does the treadmill size affect the muscle activation, i.e., with the length of the walking surface. The study aimed to investigate the influence of treadmill size, i.e., length of the walking surface, on gait pattern and to determine differences in the amplitude of muscle activation using a participant-specific musculoskeletal model (AnyBody Technology A/S, Aalborg, Denmark). For a prospective, randomized study gait parameters were collected from 47 healthy participants (aged 50.19 ± 20.58 years) while walking on two different treadmills, a small mobile treadmill (walking surface length 100 cm) and a conventional treadmill (walking surface length 150 cm), at their preferred speed, 2 km/h, and 4 km/h. Muscle activation amplitude patterns were similar between treadmills (M. gastrocnemius medialis: r_mean_ = 0.94, M. gastrocnemius lateralis: r_mean_ = 0.92, M. gluteus medius r_mean_ = 0.90, M. gluteus minimus r_mean_ = 0.94). However, the gait analysis showed a decreased preferred velocity (*p* < 0.001, *z* = 4.54), reduced stride length (preferred velocity: *p* = 0.03, *z* = −2.17; 2 km/h: *p* = 0.36, *z* = 2.10; 4 km/h: *p* = 0.006, *z* = 2.76), shorter stride time (2 km/h: *p* < 0.001, *z* = 4.65; 4 km/h: *p* < 0.001, *z* = 4.15), and higher cadence (2 km/h: *p* < 0.001, *z* = −4.20; 4 km/h: p = 0.029, *z* = −2.18) on the mobile treadmill than on the conventional treadmill. Our observations suggest that the treadmill design (e.g., a 50 cm difference in walking surface length) may not influence muscle activity amplitude during walking. However, the design of the treadmill may influence gait characteristics (e.g., stride length, cadence) of walking.

## Introduction

Locomotion is an essential movement of humans. Therefore, understanding the human gait and its influencing factors are important not only for rehabilitation but also for maintaining mobility, independence, and functioning in elderly individuals. Aging is associated with changes in both gait and the musculoskeletal system; extensive studies have shown that gait performance decreases with increasing age (1, 2). Thus, elderly persons walk slower, with reduced cadence and smaller steps, and spend more time in the double support phase of gait (2). These age-related differences can be partially explained by the loss of muscle strength and the increase in muscle activation variation (1, 2), resulting in a greater risk of falls and a higher risk of all-cause mortality (3–5). Therefore, there is a need for a solution that maintains older people's independence in a safe and efficient way, especially with increasing age (2).

There is vast knowledge regarding the influence of experimental setups, such as laboratory setups or natural environment tests, on clinical gait analysis, and the impact of different surfaces, such as instrumented treadmill or traditional laboratory walkways, on gait patterns (6–8). Compared to overground walking, the treadmill walking speed is slower and step length is reduced; furthermore, in treadmill walking, the stance and double-support phases increase while the swing duration decreases, demonstrating a “safety-related” gait adaption (9, 10). In addition, differences in joint kinematics, such as lower knee range of motion, and muscle activation patterns (lower tibialis anterior and gastrocnemius activity in the stance phase) have been observed between treadmill (11) and overground walking. Although many studies have compared treadmill walking with overground locomotion (9, 12, 13), to our knowledge there is no data on the influence treadmill design (i.e., length of the treadmill walking surface), on gait parameters, and muscle activation.

Cost-related physiotherapeutic strategies seek to attain the same level of training, but with less time or effort. Treadmill training is often used to maintain physical functioning and to train walking skills in the elderly (14). At-home (or retirement home) physical conditioning of the elderly is necessary to keep them active, leading to the development of “mobile treadmills.” These mobile devices may influence gait parameters and, consequently, result in training success. From a biomechanical perspective, McGrath et al. showed that gait speed has a significant effect on all joint movements, while different stride lengths have a more localized effect (15). Mobile treadmills have only recently appeared on the market. As only a few rehabilitation centers and hospitals have access to them, the opportunity to study gait and muscle activation on different treadmill sizes has been limited. Thus, to our knowledge, the effect of different treadmill sizes on muscle activation has not been previously reported within the scientific literature. It is reasonable to presume that a shorter treadmill will shorten the step length, but there is no evidence on the influence of changing step length on different muscle activation patterns or amplitude. Therefore, this study aimed to investigate the influence of treadmill size on gait and muscle activation amplitude. We hypothesized that the treadmill size would affect both the gait pattern (i.e., reduced step length for smaller treadmill) and therefore, lead to a reduced muscle activation amplitude.

## Materials and Methods

### Participants

Fifty participants were recruited at the Department of Orthopaedics and Trauma Surgery, University Hospital, LMU Munich. Individuals who reported orthopedic problems, joint replacement, recent traumatic injury of the lower limbs, chronic low back pain, cardiovascular and neurological diseases, or any other diseases that could negatively affect walking ability were excluded. Because of an inconsistency in the sensor calibration (possibly resulting from sensor drift due to magnetic interference), which was recognized after the measurement, three participants were excluded from this study. Therefore, 47 healthy adults were included in the analysis.

Participants were advised not to participate in any other study at the same time. All participants provided informed written consent prior to their participation. This study was approved by the ethics committee of the University Hospital of the Ludwig-Maximilian University (Project number: 17-285) and was conducted according to the principles of the Declaration of Helsinki.

### Instrumentation/Testing Equipment

**Figure 1:**
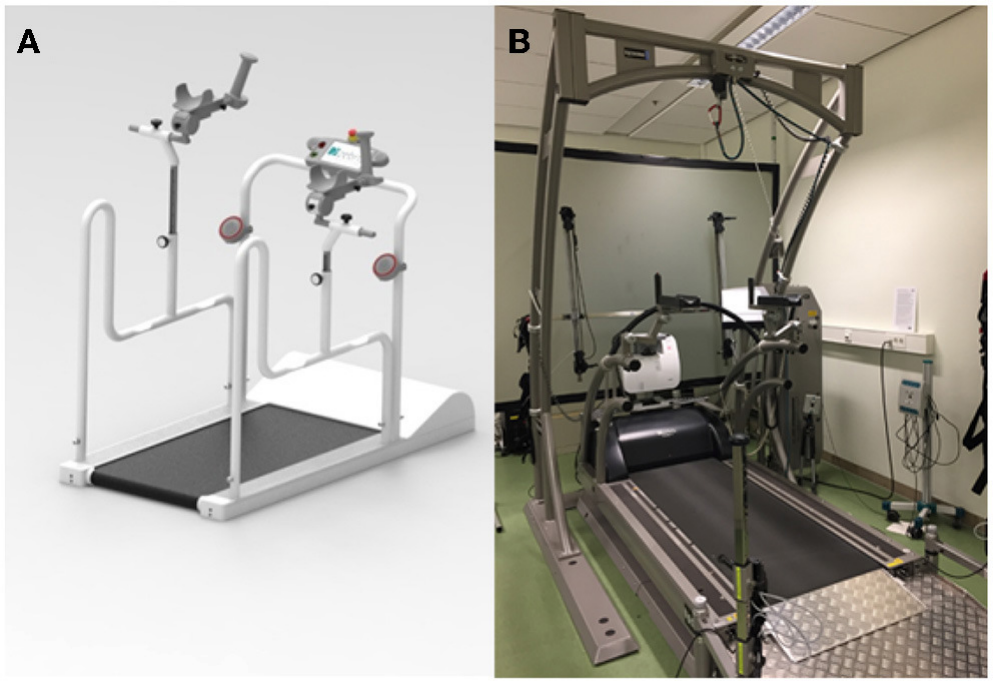
**(A)** Mobile treadmill (Zebris, Isny, Germany) with an entrance height of 5 cm, a walking surface length of 100 cm and a width of 51 cm. **(B)** Conventional treadmill (FDM-T, Zebris, Isny, Germany) with entrance height of 18 cm, a walking surface length of 150 cm and a width of 104 cm.

This study comprised two different types of treadmills. The overall picture of treadmill 1 (TM), a modified treadmill with a low entrance height of 5 cm, walking surface length of 100 cm, width of 51 cm, is shown in Figure 1; it featured a general small design, a lateral holding device, including height-adjustable armrests on both sides, and a touch display for tempo adjustments (Zebris, Isny, Germany). Treadmill 2 was an instrumented treadmill, featuring a ramp, lateral and frontal holding devices, and integrated measuring sensor matrix/integrated force and pressure sensors (FDM-T, Zebris, Isny, Germany), with an entrance height of 18 cm, a walking surface length of 150 cm and a width of 104 cm (Figure 1).

Spatiotemporal gait parameters were collected using an inertial motion capture system with wearable motion trackers (Xsens Technologies, Enschede, Netherlands) integrating three-dimensional (3D) gyroscopes, a 3D accelerometer, and 3D magnetometers and at a sampling frequency of 60 Hz. To measure full body kinematics, 15 motion trackers were attached to the following body parts: feet, lower legs, upper legs, shoulders, upper arms, lower arms, pelvis, sternum, and head. Data of each motion tracker were collected in Xsens MVN Software, transferred to a wireless access point (Awinda Station, Xsens), and connected to a laptop.

### Experimental Design

**Figure 2:**
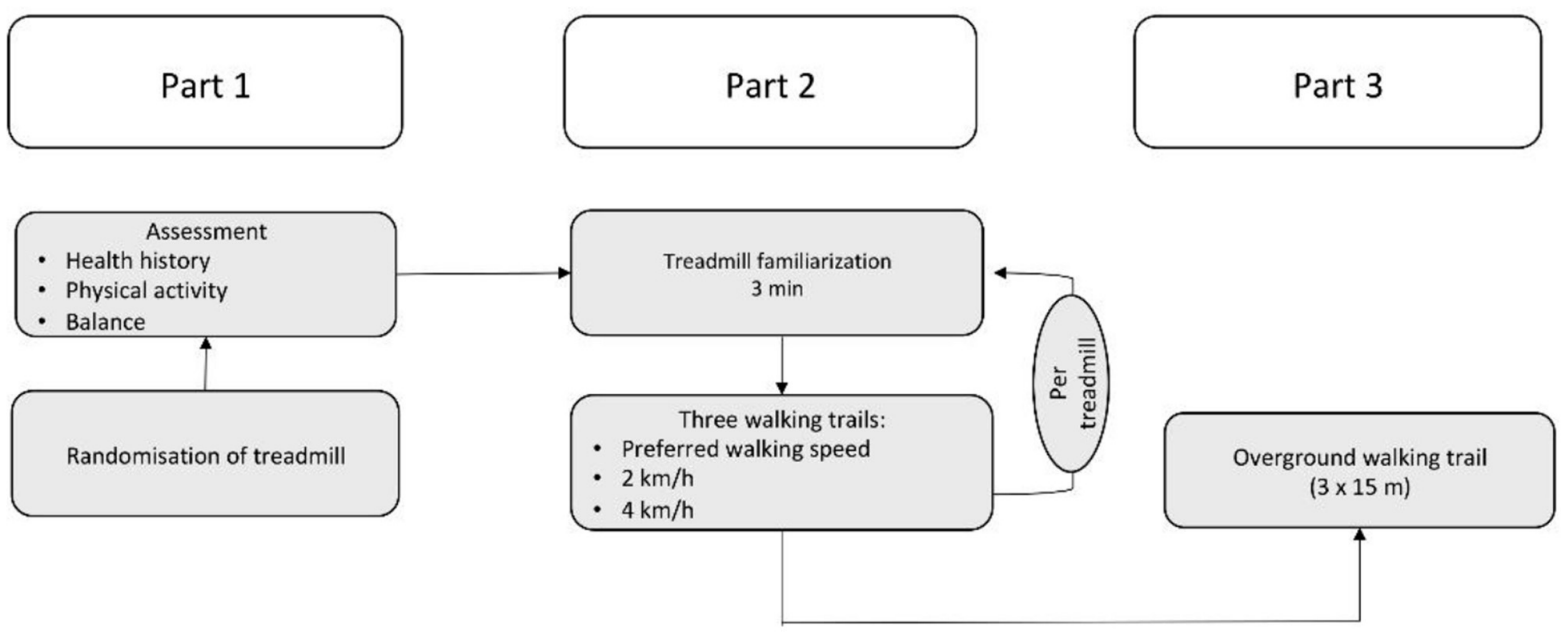
Experimental Design. Part 1: Assessment of health history, physical activity status, and balance. Afterwards, participants were randomly allocated either treadmill. Part 2: Participants had a familiarization time of 3 min on treadmill 1; thereafter, gait parameters were assessed at their preferred walking speed, 2 and 4 km/h. Part 3: At the end of the test, gait parameters were assessed during overground walking.

The study procedure is shown in Figure 2. Participants were randomized to each treadmill (starting treadmill). All participants completed one test session, consisting of three parts. In the first part, the physical fitness level, balance ability, and health history of the participants were assessed. In the second part, participants, equipped with Xsens, performed three walking trials at different speeds. Participants were blinded to the speed display and were not allowed to hold onto the armrests while walking on the treadmill to avoid possible disturbances in the gait pattern. At first, the preferred walking speed (PWS) of the participant, defined as a self-selected or comfortable gait speed (9), was determined by increasing the speed by 0.1 km/h every three steps until participants reported difficulties in maintaining the walking speed. Participants were allowed to familiarize themselves with walking on the treadmill at their PWS for 3 min. After familiarization, participants were allowed to adjust the PWS. After determination of the PWS, gait data were collected for 30 sec. Subsequently, participants were asked to adjust their speed to 2 and 4 km/h, and each gait was measured for 30 s. Participants were then asked to manually stop the treadmill and step down. The same procedure was used for the second treadmill. In the third part, the overground walking speed was measured. Participants were asked to walk on a 15 m walkway at their PWS three times. The average overground speed was calculated from the three trials.

### Assessments

#### Musculoskeletal Model

In this study, the primary outcome was the muscle activation amplitude. To analyze the activation pattern of the amplitude of lower limb muscles, a musculoskeletal model was created in the AnyBody Modeling System (AMS) v.7.2 (AnyBody Technology A/S, Aalborg, Denmark) for 15 participants, whose demographic data (i.e., age, weight and height) were matched to the mean demographic data of the initial 47 participants. The AnyMOCAP base model from the AnyBody Managed Model Repository (AMMR) v.2.1.1 was used to simulate the musculoskeletal model based on the Xsens Data (BVH = Biovision Hierarchy file). The use of AnyBody to calculate muscle forces based on motion data has been well-validated (16–18). As a first step, a basic model was programmed using subject-specific body height, weight, and dimensions from the Xsens Data. Experiment marker trajectories were tracked by the model's marker; thus body segments and joint positions could be detected (19). Because the ground reaction force (GRF) was not measured in the experiment, a GRF prediction in the model was necessary. Therefore, the modeling system uses its own GRF prediction algorithm, using contact nodes on the bottom of the foot. This algorithm has been validated and applied in several studies (20, 21). Thereafter, an inverse dynamic calculation was performed in the AnyBody Modeling System to obtain the muscle activation amplitude. The modeling of the muscles was based on Hill's three component muscle model, which influences muscle strength (22). Muscle activation was defined as the muscle force divided by the strength of the muscle. The total body model consists among others of the lower body model, which contains 110 muscles, divided into 318 individual muscle strands (23). For this study, the following muscles were examined more closely in the later evaluation: M. gluteus maximus, M. gluteus medius, M. gluteus minimus, M. vastus lateralis, M. vastus medialis, M. biceps femoris, M. semitendinosus, M. gastrocnemius lateralis, and M. gastrocnemius medialis.

#### Gait Analysis

Spatiotemporal gait parameters (gait velocity and stride length) were assessed using the inertial motion capture system (Xsens Technologies, Enschede, Netherlands). These parameters were measured for each treadmill walking trial. Stride length was calculated for all steps of the whole measuring time of 30 s for treadmill walking. For overground walking gait data of the entire 15 m was used to calculate stride length.

#### Physical Activity Level

The physical activity level of the participants was estimated using the German short version of the International Physical Activity Questionnaire (IPAQ). The IPAQ short form is widely used and evaluates three types of activities: vigorous activity, moderate activity, and walking. The duration and frequency were collected separately for each activity. This questionnaire sorts/classifies individuals into the following categories: low (score 1), moderate (score 2), and high (score 3) active (24). The data analysis was performed according to the published guidelines (25).

#### Balance Confidence Scale

Balance confidence, or confidence to perform activities without falling, was evaluated using the German version of the Activities-Specific Balance Confidence (ABC) scale (26). This validated scale consists of 16 activities of daily living with varying degrees of difficulty. The balance confidence scale ranges from 0% (low) to 100% (high). A score <50% indicates low balance confidence, and a score between 50 and 80% shows moderate balance confidence. Healthy athletic adults score more than 80% on average (27, 28).

### Statistical Analyses

Descriptive statistics are presented as the mean and standard deviation and were used to describe gait speed and step length. All statistical analyses were performed using SPSS version 25 (IBM Corp., Armonk, NY, USA). The data was subjected to the Kolmogorov-Smirnov test of normality and failed. Therefore, a Wilcoxon test was used to compare differences in velocity, stride length, and gait performance. A *p*-value of < 0.05 was considered statistically significant.

Further analyses of the muscle activation were performed via MATLAB R2019a (MathWorks, Inc., Natick, MA, USA). The muscle activation from treadmill 1 and treadmill 2 was normalized to 100% gait cycle (one double step), and the average was calculated for each relevant muscle of each participant. Further, the respective muscle activation from treadmill 1 and treadmill 2 was compared by applying Pearson's correlation (r_mean_) to the maximum activation observed in each participant on each treadmill.

## Results

Descriptive characteristics of the 47 participants are summarized in Table 1. No adverse or harmful events were reported during any part of the experiment.

**Table 1:** Demographic and clinical information of the participants and the subgroup for the AnyBody model.

**Demographic information**	**Total sample,**	**Subgroup, anybody**
	**mean (±SD), *N* = 47**	**model mean**
		**(±SD), *N* = 15**
Sex (m/f)	6/41	3/12
Age (years)	50.19 (± 20.58)	51.57 (± 21.02)
Height (cm)	167.51 (± 8.59)	169.93 (± 8.22)
Weight (kg)	64.21 (± 9.87)	65.93 (± 10.38)
Body mass index (kg/m^2^)	22.93 (± 3.43)	22.83 (± 0.15)
**Clinical information**
IPAQ*	2.48 (± 0.50)	
ABCD**(%)	96.97 (± 3.75)	

* *IPAQ: International Physical Activity Questionnaire (1 = low activity, 2 = moderate activity, 3 = high activity)*.

** *ABCD: Balance Confidence Scale (≤ 50% low confidence, 50–80% moderate balance confidence)*.

*Demographic information includes age, body mass index, height, and weight. Clinic information includes International Physical Activity Questionnaire (IPAQ) and Balance Confidence Scale (ABCD) score*.

### Muscle Activation Amplitude

Based on Lee and Hidler a sample size calculation was done for the musculoskeletal model by the maximum hip extension moment, resulting in a sample size of *n* = 14 (11). Therefore, the musculoskeletal model was created for 15 participants. They were selected to reflect the demographic data of the total population in age, weight, and height (Table 1).

**Figure 3:**
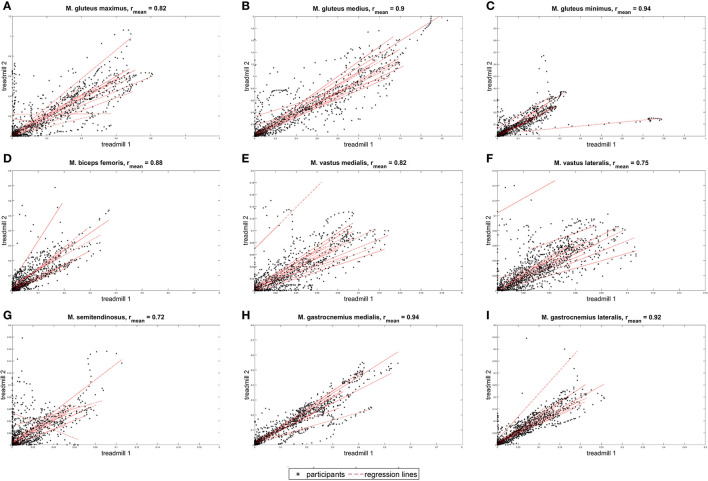
Correlation of the different muscle activation patterns. Nine graphs showing the correlation of different muscle activations between the mobile (treadmill 1) and conventional treadmills (treadmill 2). Arrangement from left to right: **(A)** M. gluteus maximus, **(B)** M. gluteus medius, **(C)** M. gluteus minimus, **(D)** M. biceps femoris, **(E)** M. vastus medialis, **(F)** M. vastus lateralis, **(G)** M. semitendinosus, **(H)** M. gastrocnemius medialis, and **(I)** M. gastrocnemius lateralis. The unit is [muscle force/muscle strength].

The muscle activation amplitude showed high correlations (Figure 3) between 0.94 for M. gastrocnemius medialis and 0.72 for M. semitendinosus. The maximal activation of the different muscles calculated by the AnyBody Modeling System showed only small differences (max. Activation treadmill 2–max. Activation treadmill 1) between −0.02 (SD −0.08) and 0.11 (SD 0.03) (Table 2).

**Table 2:** Difference in maximal muscle activations between the conventional treadmill and the small treadmill, the unit is muscle force/muscle strength (*n* = 15).

**Muscle**	**Mean difference (treadmill 2–treadmill 1) of**
	**maximal muscle activation (±SD)**
M. gluteus maximus	0.04 (± 0.01)
M. gluteus medius	0.02 (± 0.12)
M. gluteus minimus	–0.02 (± –0.08)
M. gastrocnemius lateralis	0.03 (± 0.00)
M. biceps femoris	0.11 (± 0.03)
M. vastus lateralis	0.04 (± –0.01)
M. gastrocnemius medialis	0.00 (± 0.01)
M. semitendinosus	0.02 (± 0.01)
M. vastus medialis	0.06 (± 0.00)

### Gait Patterns

Since stride length is dependent on body height, the values are standardized as follows: stride length [cm]^*^(individual height [cm]/mean height [cm]). This is also the case for cadence: cadence [steps/min] ^*^ (individual height [cm]/mean height [cm]).

#### Overground vs. Treadmill 1 and Overground vs. Treadmill 2

All 47 participants were included in the comparison of the gait parameters between overground walking and treadmill 1 as well as between overground walking and treadmill 2. The preferred overground velocity was significantly higher than that on treadmill 1 or treadmill 2 (overground = 5.17 km/h, treadmill 1 = 2.89 km/h, *p* < 0.001, *z* = −5.97; treadmill 2 = 3.46 km/h; *p* < 0.001, *z* = −5.87). The results revealed a significantly wider stride length during overground walking at the preferred speed (overground = 100.6 cm, treadmill 1 = 76.8 cm, *p* < 0.001, *z* = −4.12; treadmill 2 = 80.2 cm, *p* = 0.008, *z* = −2.66) and a significant increase in overground cadence compared with that on treadmill 1 and treadmill 2 (overground = 110.5 steps/min, treadmill 1 = 99.2 steps/min, *p* < 0.001, *z* = −4.73; treadmill 2 = 99.1, *p* < 0.001, *z* = −4.71). Additionally, a shorter overground stride time was observed compared to that of treadmill 1 and treadmill 2 when walking at the preferred speed (overground = 1.3 s, 1 treadmill = 1.17 s, *p* < 0.001, *z* = 5.24; 2 treadmill = 1.14 s, *p* < 0.001, *z* = 5.23). Furthermore, the results show a significant difference in the stance and swing phases for the preferred walking speed in overground walking compared with that in treadmill 1 (*p* < 0.001) and treadmill 2 (*p* < 0.001), revealing a longer stance phase and a shorter swing phase on both treadmills.

#### Treadmill 1 vs. Treadmill 2

All 47 participants were included in the comparison between treadmills 1 and 2. Among the total participants, the preferred velocity on treadmill 2 (3.46 km/h) was significantly higher than that on treadmill 1 (2.89 km/h) (*p* < 0.001, *z* = 4.54). The stride length at 2 and 4 km/h was significantly longer on treadmill 2 than that on treadmill 1 (2 km/h: treadmill 1 = 64.6 cm, treadmill 2 = 69.2 cm, *p* = 0.36, *z* = 2.10; 4 km/h: treadmill 1 = 88.0, treadmill 2 = 99.5 cm, *p* = 0.006, *z* = 2.76). However, when walking at 2 km/h and 4 km/h, the cadence was higher on treadmill 1 than on treadmill 2 (2 km/h: treadmill 1 = 76.6 steps/min, treadmill 2 = 70.9 steps/min, *p* < 0.001, *z* = −4.20; 4 km/h: treadmill 1 = 104.5 steps/min, treadmill 2 = 101.3 km/h, *p* = 0.029, *z* = −2,18). The results revealed a significantly shorter stride time on treadmill 1 than that on treadmill 2 at 2 km/h (treadmill 1 = 1.48 sec, treadmill 2 = 1.60 s, *p* < 0.001, *z* = 4.65) and 4 km/h (treadmill 1 = 1.07 s, treadmill 2 = 1.12 s, *p* < 0.001, *z* = 4.15). In addition, a significant difference was found in the swing and stance phases for the preferred walking speed at 4 km/h (*p* = 0.657), but not at 2 km/h (*p* < 0.001) in treadmills.

## Discussion

This study investigated the influence of treadmill design/size on muscle activation amplitude and gait of the lower extremities. The major finding of this study was that the length of the treadmill does not influence muscle activation amplitude and maximal muscle activation, since muscle activation amplitude appeared to be the same on both treadmill designs as indicated by the significant positive correlations of different muscle groups.

Since studies have shown that not only gait patterns but also muscle activation patterns change with age (2, 29, 30), there is a need for a safe and efficient solution to restore and train these muscle functions. Although there were differences in gait patterns between overground and treadmill walking and between the treadmill designs, no differences were observed in muscle activation amplitude itself which is also in agreement with Mazaheri et al. (31). This provides a first initial evidence for comparable muscle activation but needs further examination on how the central nervous system will respond to these different environment situations (mobile = more restrained environment vs. conventional = less restrained environment). Mileti et al. have shown that that different environment restrains might forced the CNS to adopt a different neural control strategy (32).

Muscle weakness is often found among the elderly population, and besides gait and balance deficits, it is the most important risk factor for falls (33). Globally, 28 to 35% of people aged 65 years and older report a fall, annually, followed by consequences, such as traumatic injuries and long hospital stays (34). In particular, people living in nursing homes fall more often than people living in the community (34), showing that nursing homes are also a risk factor for falls (35). To address this, the small and mobile treadmill with a low entrance height could be used in nursing homes. Our results give a first evidence that the use of a small mobile treadmill seems appropriate for at-home training, since no benefit loss was observed. Accompanied with the benefit of more muscle function (higher amplitude) when walking on a treadmill, treadmill training would especially be useful for people with core muscle weakness (31).

Compared with overground walking, the gait analysis revealed a significantly lower preferred walking speed, decreased cadence, shorter stride length, longer stride time, longer stance phase, and shorter swing phase on both treadmills. These adaptations in the gait pattern is in line with the results of previous studies (13, 36). The participant's sense of security on the treadmill was reduced, which was reflected by the safety-related gait adaptation/cautious gait (9, 36). In addition, the difference in gait parameters on the smaller treadmill (i.e., longer stance phase) could result from the lower PWS (37).

The analysis of the gait parameters between both treadmills showed a reduced stride length, shorter stride time, and higher cadence on the mobile treadmill compared to the conventional treadmill. This gait adaptation could be explained by the influence of the treadmill length. The short treadmill design limits the stride length; to maintain the given speed (2 or 4 km/h), a reduction of stride time and an increase in cadence were necessary. To our knowledge, since this is the first study investigating this field, there is no other data to compare this result with.

Our study has some limitations that should be addressed and considered when interpreting the results. First, a familiarization period of only 3 min was chosen for each treadmill. Most of the participants showed problems in maintaining or reaching a stable walk during the 3-min familiarization period on the small treadmill, leading to an unnatural gait. Until recently, a guideline of the acclimatization time has been lacking. Several studies suggest different time periods ranging from 4 to 6 min and up to 15 min for older individuals (38). A recent study revealed that a minimum acclimatization time of 6 min is necessary to reach the acclimatization (familiarization) plateau for all parameters but for parameters like step length 224 s are needed (38). Our protocol included a PWS selection (~90 s) before the acclimatization time of 3 min at PWS. Therefore, we have chosen a 3-min familiarization time which in total means a acclimatization time for the treadmill walk of ~270 s in this study but perhaps this was too short and a longer familiarization time might be preferable in further studies. Second, our study population might not be representative of the general population, since most of our participants were active and had a positive attitude toward physical activity. This might lead to a higher physical activity level of the participants. Additionally, the majority of the participants were women, restricting generalization, and the wide age group (adult to elderly) led to a greater standard deviation in the age of the participants. Third, given the high complexity of developing musculoskeletal computer model, they were only used in 15 participants. Fourth, the musculoskeletal computer model itself was a limitation in this study. Although this tool is validated in the literature, the gold standard for measuring muscle activation is electromyography. Furthermore, the small walking surface of the mobile treadmill could lead to walking difficulties, such as the short stride length found in this study. Fourth, GRF was not measured directly and was calculated via a GRF prediction within the Anybody Modeling Software. This GRF prediction is a validated procedure and showed a high correlation with am mean person correlation of 0.957 (20). Fifth, the high cost of the newly developed mobile treadmills seems to be a relevant disadvantage, which may restrict access of the general population.

In conclusion, this study provides first insights into gait patterns and muscle activation on different treadmill designs/sizes to complement the known gait characteristics and muscle activation on treadmills. Muscle activation amplitude appeared to be the same on both treadmills, although differences in gait parameters were recognized. These results provide the basis for individual physical muscle training on small mobile treadmill designs, which might be used at home or similar outpatient settings. Further research is needed to assess the long-term effects of training with a mobile treadmill and in detail the influence of such a system on the muscle activation pattern.

## Data Availability Statement

The datasets generated for this study will not be made publicly available due to German legislation. Questions regarding the datasets can be sent to matthias.woiczinski@med.uni-muenchen.de.

## Ethics Statement

The studies involving human participants were reviewed and approved by Ludwig-Maximilian University. The patients/participants provided their written informed consent to participate in this study.

## Author Contributions

MW and CL analyzed the data, interpreted the results, drafted the manuscript, participated in the planning of the study, designing the intervention protocols, and the participant recruitment. TE and MA carried out the recruitment, assessed the eligibility of the participants, and contributed to the interpretation of the results. JL participated in planning the study and revision of the manuscript. MK and LB contributed to the analysis and the interpretation of the results. EK supervised the study, interpreted the results, and revised the manuscript. All authors approved the manuscript to be submitted.

## Funding

This research was funded by Central Innovation Programme 760 for small and medium-sized enterprises (SMEs) Germany 761 (ZF4270101AK6) in cooperation with Zebris.

## Conflict of Interest

This research was funded by Central Innovation Programme for small and medium-sized enterprises (SMEs) Germany (ZF4270101AK6) in cooperation with Zebris Medical GmbH. The authors declare that the research was conducted in the absence of any commercial or financial relationships that could be construed as a potential conflict of interest.

## Publisher's Note

All claims expressed in this article are solely those of the authors and do not necessarily represent those of their affiliated organizations, or those of the publisher, the editors and the reviewers. Any product that may be evaluated in this article, or claim that may be made by its manufacturer, is not guaranteed or endorsed by the publisher.
